# Chromosomal and DNA barcode analysis of the *Melitaea ala* Staudinger, 1881 species complex (Lepidoptera, Nymphalidae)

**DOI:** 10.3897/CompCytogen.v15.i2.66121

**Published:** 2021-06-18

**Authors:** Vladimir A. Lukhtanov, Anastasia V. Gagarina, Elena A. Pazhenkova

**Affiliations:** 1 Department of Karyosystematics, Zoological Institute of the Russian Academy of Sciences, Universitetskaya nab. 1, St. Petersburg 199034, Russia Zoological Institute of the Russian Academy of Sciences St. Petersburg Russia; 2 Department of Entomology, St. Petersburg State University, Universitetskaya nab. 7/9, St. Petersburg 199034, Russia St. Petersburg State University St. Petersburg Russia

**Keywords:** chromosome, *COI*, DNA barcode, karyosystematics, *
Melitaea
*, taxonomy

## Abstract

The species of the *Melitaea ala* Staudinger, 1881 complex are distributed in Central Asia. Here we show that this complex is a monophyletic group including the species, *M. ala*, *M. kotshubeji* Sheljuzhko, 1929 and *M. enarea* Fruhstorfer, 1917. The haploid chromosome number n=29 is found in *M. ala* and *M. kotshubeji* and is, most likely, a symplesiomorphy of the *M. ala* complex. We show that *M. ala* consists of four subspecies: *M. ala zaisana* Lukhtanov, 1999 (=*M. ala irtyshica* Lukhtanov, 1999, **syn. nov.**) (South Altai, Zaisan Lake valley), *M. ala ala* (Dzhungarian Alatau), *M. ala bicolor* Seitz, 1908 (North, East, Central and West Tian-Shan) and *M. ala determinata* Bryk, 1940 (described from “Fu-Shu-Shi”, China). We demonstrate that *M. kotshubeji kotshubeji* (Peter the Great Mts in Tajikistan) and *M. kotshubeji bundeli* Kolesnichenko, 1999 (Alai Mts in Tajikistan and Kyrgyzstan) are distinct taxa despite their geographic proximity in East Tajikistan. *Melitaea enarea* is widely distributed in the southern part of Central Asia and is sympatric with *M. kotshubeji*.

## Introduction

This work is a continuation of a series of publications (Lukhtanov and Kuznetsova 1989; Pazhenkova et al. 2015; Pazhenkova and Lukhtanov 2016; Lukhtanov 2017) devoted to the analysis of chromosomal and mitochondrial haplotype diversity and taxonomy of butterflies of the species-rich butterfly genus *Melitaea* Fabricius, 1807. The combination of molecular and cytogenetic methods is a useful tool for taxonomic studies (Lukhtanov et al. 2015; Pazhenkova and Lukhtanov 2019) and can be a good addition to morphological analysis of taxonomically complicated groups of species (Lukhtanov et al. 2016). In our previous papers, we applied analysis of the DNA barcodes and karyotypes to study the genetic and taxonomic structure of the *M. didyma* (Esper, 1779) (Pazhenkova et al. 2015; Pazhenkova and Lukhtanov 2016) and *M. persea* Kollar, 1849 (Lukhtanov 2017) species complexes. The aim of this work is to study a complex of species close to *M. ala* Staudinger, 1881.

The species of this complex are distributed in Central Asia (Kolesnichenko 1999). According to Kolesnichenko (1999), this complex consists of the following species: *Melitaea ala* Staudinger, 1881, *M. kotshubeji* Sheljuzhko, 1929, *M. ninae* Sheljuzhko, 1935, *M. chitralensis* Moore, 1901, and *M. enarea* Fruhstorfer, 1917. According to van Oorschot and Coutsis (2014), this complex consists of the following species: *M. acraeina* Staudinger, 1881, *M. ninae* Sheljuzhko, 1935, *Melitaea ala* Staudinger, 1881, *M. didymina* Staudinger, 1895, *M. chitralensis* Moore, 1901, *M. enarea* Fruhstorfer, 1917, *M. bundeli* Kolesnichenko, 1999, *M. kotshubeji* Sheljuzhko, 1929, *M. sutschana* Staudinger, 1881 and *M. yagakuana* Matsumura, 1927 (the latter taxon is usually considered a subspecies of *M. sutschana*, e.g. see Higgins, 1941).

Molecular phylogenetic analysis (Leneveu et al. 2009) demonstrated that *M. ala* and *M. enarea* (cited in the article as *M. permuta* Higgins, 1941) are sister species, and *M. acraeina* is a phylogenetically distant species which is a sister to the lineage (*M. ala* + *M. enarea*). *Melitaea sutschana* was found as a member of the *M. didyma* species complex which is a sister to the lineage ((*M. acraeina* + (*M. ala* + *M. enarea*)) (Leneveu et al. 2009). In our study, we focused on the analysis of the *M. ala* lineage. We did not include *M. ninae*, *M. didymina* and *M. chitralensis* in the analysis, since for these species there has been no material suitable for chromosomal and molecular studies.

## Materials and methods

### Chromosomal analysis

Karyotypes of four samples of *M. kotshubeji kotshubeji* were studied as previously described (Lukhtanov et al. 2014; Vishnevskaya et al. 2016). Briefly, gonads were removed from the adult male abdomen and placed into freshly prepared fixative (3:1; 96% ethanol and glacial acetic acid) directly after capturing the butterfly in the field. Testes were stored in the fixative for 3–36 months at +4 °C. Then the gonads were stained in 2% acetic orcein for 5–10 days at +18–20 °C. Different stages of male meiosis, including metaphase I (MI) and metaphase II (MII) were examined using an original two-phase method of chromosome analysis (Lukhtanov et al. 2006, 2008). Leica DM2500 light microscope equipped with HC PL APO 100×/1.44 Oil CORR CS lens and S1/1.4 oil condenser head was used for bright-field microscopy analysis. A Leica DM2500 light microscope equipped with HC PL APO 100×/1.40 OIL PH3 lens was used for phase-contrast microscopy analysis.

### Molecular methods and DNA barcode analysis

Standard *COI* barcodes (658-bp 5’ fragment of *mitochondrial cytochrome oxidase subunit I*) were studied as previously described (Lukhtanov et al. 2014; Vishnevskaya et al. 2016). *COI* sequences were obtained from 34 specimens representing the *M. ala* species group and outgroups (*M. telona* Fruhstorfer, 1908 and *M. alatauica* Staudinger, 1881). Legs were used as a source for DNA isolation

Legs from 6 specimens (*M. kotshubeji bundeli* Kolesnichenko, 1999) were processed in the Department of Karyosystematics of Zoological Institute of the Russian Academy of Sciences using primers and protocols described by Shapoval et al. (2017). Sequencing was carried out at the Research Resource Center for Molecular and Cell Technologies of St. Petersburg State University.

Legs from 28 specimens of *Melitaea* spp. were processed in the the Canadian Centre for DNA Barcoding (**CCDB**, Biodiversity Institute of Ontario, University of Guelph) using their standard high-throughput protocol described by Hajibabaei et al. (2005), Ivanova et al. (2006) and deWaard et al. (2008). The set of voucher specimens of butterflies is kept in the Zoological Institute of the Russian Academy of Science (St. Petersburg) and in the McGuire Center for Lepidoptera and Biodiversity (**MGCL**), Florida Museum of Natural History, University of Florida, Gainesville, Florida, USA. Photographs of these specimens, as well as collecting data are available in the of Life Data System (BOLD), projects Butterflies of Palearctic (BPAL) and Butterflies of Palearctic Part B (BPALB) at http://www.boldsystems.org/.

We also used 30 published *COI* sequences for DNA barcode analysis (Leneveu et al. 2009; Lukhtanov et al. 2009; Ashfaq et al. 2013; Pazhenkova et al., 2015; Pazhenkova and Lukhtanov 2016; Lukhtanov 2017) (Table 1).

**Table 1.** Specimens of *Melitaea* spp. used in the DNA barcode analysis.

Species and subspecies	Species name as found in GenBank	Field code or BOLD number	GenBank number	Country	Locality	Reference
*M. acentria*	*M. acentria*	BOLD:BPAL2191-13	**KY777529**	Israel	Hermon	Lukhtanov 2017
*M. acraeina*	*M. acraeina*	BOLD:GBLN1879-09	**FJ462229**	Uzbekistan	Komsomolobad	Leneveu et al. 2009
*M. ala ala*	*M. ala*	BPALB179-16; CCDB-25458_G12	**MW672072**	Kazakhstan	Dzhungarian Mts, Kopal, 45.08°N, 79.07°E	This study
*M. ala ala*	*M. ala*	BOLD:BPAL039-10	**MW672074**	Kazakhstan	Taldy-Kurgan region, Kysylagash	This study
*M. ala ala*	*M. ala*	BOLD:BPAL3407-16	**MW672077**	Kazakhstan	Taldy-Kurgan region, Kyzylagash	This study
*M. ala bicolor*	*M. ala*	BOLD:GBLN1877-09	**FJ462231**	China	Tian-Shan	Leneveu et al. 2009
*M. ala bicolor*	*M. ala bicolor*	BOLD:LOWA355-06	**FJ663775**	Kyrgyzstan	Moldatoo Mts, 41.5°N, 74.62°E	Lukhtanov et al. 2009
*M. ala bicolor*	*M. ala bicolor*	BOLD:LOWA356-06	**FJ663774**	Kyrgyzstan	Moldatoo Mts, 41.5°N, 74.62°E	Lukhtanov et al. 2009
*M. ala bicolor*	*M. ala bicolor*	BOLD:BPAL2288-14	**MW672075**	China	Xinjiang, Kunges Valley	This study
*M. ala bicolor*	*M. ala bicolor*	BOLD:BPAL2289-14	**MW672076**	China	Xinjiang, Kunges Valley	This study
*M. ala bicolor*	*M. ala bicolor*	BOLD:BPAL012-10	**MW672079**	Kazakhstan	Kirgizsky Mts, Kaindy	This study
*M. ala bicolor*	*M. ala bicolor*	BOLD:BPAL013-10	**MW672080**	Kazakhstan	Kirgizsky Mts, Kaindy	This study
*M. ala bicolor*	*M. ala bicolor*	BOLD:BPAL026-10	**MW672081**	Kyrgyzstan	Talassky Mts, Kara-Bura Pass	This study
*M. ala bicolor*	*M. ala bicolor*	BOLD:BPAL027-10; RPVL-00027	**MW672082**	Kyrgyzstan	Talassky Mts, Kara-Bura Pass	This study
*M. ala bicolor*	*M. ala bicolor*	BOLD:BPAL3499-16	**MW672089**	Kyrgyzstan	Talassky Mts, Kara-Bura Pass	This study
*M. ala bicolor*	*M. ala bicolor*	BOLD:BPAL3500-16	**MW672090**	Kyrgyzstan	Talassky Mts, Kara-Bura Pass	This study
*M. ala bicolor*	*M. ala bicolor*	BOLD:BPAL3501-16	**MW672091**	Kyrgyzstan	Talassky Mts, Kara-Bura Pass	This study
*M. ala bicolor*	*M. ala bicolor*	BOLD:BPAL009-10; CCDB-03024-RPVL-00009	**MW672078**	Kazakhstan	Kirgizsky Mts, Merke River	This study
*M. ala irtyshica*	*M. ala*	BOLD:BPALB181-16	**MW672073**	Kazakhstan	Zyryanovsk region, 49.62°N, 83.62°E	This study
*M. ala irtyshica*	*M. ala*	BOLD:BPAL3481-16	**MW672083**	Kazakhstan	Zyryanovsk region, 49.62°N, 83.62°E	This study
*M. ala irtyshica*	*M. ala*	BOLD:BPAL3483-16	**MW672085**	Kazakhstan	Zyryanovsk region, 49.62°N, 83.62°E	This study
*M. ala irtyshica*	*M. ala*	BOLD:BPAL3484-16; CCDB-25456_F04	**MW672086**	Kazakhstan	Zyryanovsk region, 49.62°N 83.62°E	This study
*M. ala irtyshica*	*M. ala*	BOLD:BPAL3485-16	**MW672087**	Kazakhstan	Zyryanovsk region, 49.62°N, 83.62°E	This study
*M. ala irtyshica*	*M. ala*	BOLD:BPAL3486-16	**MW672088**	Kazakhstan	Zyryanovsk region, 49.62°N 83.62°E	This study
*M. ala zaisana*	*M. ala zaisana*	BOLD:LOWA174-06	**FJ663777**	Kazakhstan	Kurchumski Khrebet 48.47°N, 84.12°E	Lukhtanov et al. 2009
*M. ala zaisana*	*M. ala zaisana*	BOLD:LOWA175-06	**FJ663776**	Kazakhstan	Kalgutynski Pass, 48.47°N 84.12°E	Lukhtanov et al. 2009
*M. alatauica*	*Mellicta alatauica*	BOLD:PALB177-16	**MW672064**	Kazakhstan	Dzhungarian Mts, Kopal, 45.08°N, 79.07°E	This study
*M. alatauica*	*Mellicta alatauica*	BOLD:LOWA273-06	**FJ663811**	Kazakhstan	Dshungarski Alatau, Koksu, 44.72°N, 79.0°E	Lukhtanov et al. 2009
*M. alatauica*	*Mellicta alatauica*	BOLD:LOWA274-06	**FJ663810**	Kazakhstan	Dshungarski Alatau, Koksu, 44.72°N, 79.0°E	Lukhtanov et al. 2009
*M. casta*	*M. casta*	BOLD:BPAL2306-14	**KY777552**	Iran	Lorestan	Lukhtanov 2017
*M. deserticola*	*M. deserticola*	BOLD:BPAL3124-15	**KY086157**	Israel	Jerusalem	Pazhenkova and Lukhtanov 2016
*M. didyma*	*M. didyma*	BOLD:BPAL2495-14	**KT874733**	Austria	Tirol	Pazhenkova et al. 2015
*M. didymoides*	*M. didymoides*	BOLD:BPAL3493-16	**KY086178**	Russia	Buryatia	Pazhenkova and Lukhtanov 2016
*M. enarea*	*M. enarea*	BOLD:BPAL2656-14	**MW672065**	Tajikistan	Tabakchi, 37.85° N, 68.98°E, 1200 m	This study
*M. enarea*	*M. enarea*	BOLD:BPAL2657-14	**MW672066**	Tajikistan	Chaltau, 37.9550°N, 69.1403°E; 1041m	This study
*M. enarea*	*M. enarea*	BOLD:BPAL2658-14	**MW672067**	Tajikistan	Chaltau, 37.9550°N, 69.1403°E; 1041m	This study
*M. enarea*	*M. enarea*	BOLD:BPAL2659-14; CCDB-17967_H10	**MW672068**	Tajikistan	Chaltau, 37.9550°N, 69.1403°E; 1041m	This study
*M. enarea permuta*	*M. enarea permuta*	BOLD:GBLN1837-09	**FJ462272**	Uzbekistan	Ghissar Mts	Leneveu et al. 2009
*M. gina*	*M. gina*	BOLD:BPAL3083-15	**KY086152**	Iran	35.32°N, 47.15°E	Pazhenkova and Lukhtanov 2016
*M. higginsi*	*M. higginsi*	BOLD:BPAL2469-14	**KY777548**	Afghanistan		Lukhtanov 2017
*M. interrupta*	*M. interrupta*	BOLD:BPAL3019-15	**KY086139**	Georgia	Bakuriani	Pazhenkova and Lukhtanov 2016
*M. kotshubeji bundeli*	*Melitaea ala bundeli*	GA161	**MW672092**	Tajikistan	Alai Mts, 39.42°N, 71.62°E	This study
*M. kotshubeji bundeli*	*Melitaea ala bundeli*	GA162	**MW672093**	Tajikistan	Alai Mts, 39.42°N, 71.62°E	This study
*M. kotshubeji bundeli*	*Melitaea ala bundeli*	GA163	**MW672094**	Tajikistan	Alai Mts, 39.42°N, 71.62°E	This study
*M. kotshubeji bundeli*	*Melitaea ala bundeli*	GA164	**MW672095**	Tajikistan	Alai Mts, 39.42°N, 71.62°E	This study
*M. kotshubeji bundeli*	*Melitaea ala bundeli*	GA165	**MW672096**	Tajikistan	Alai Mts, 39.42°N, 71.62°E	This study
*M. kotshubeji bundeli*	*Melitaea ala bundeli*	GA166	**MW672097**	Tajikistan	Alai Mts, 39.42°N, 71.62°E	This study
*M. kotshubeji kotshubeji*	*M. ala kotshubeji*	BOLD:BPAL2308-14	**MW672069**	Tajikistan	Peter I Range, Garm	This study
*M. kotshubeji kotshubeji*	*M. ala kotshubeji*	BOLD:BPAL2484-14; CCDB-17966 B02	**MW672070**	Tajikistan	Peter I Range, 7 km S Tajikobad	This study
*M. kotshubeji kotshubeji*	*M. ala kotshubeji*	BOLD:BPAL2485-14	**MW672071**	Tajikistan	Peter I Range, Garm	This study
*M. latonigena*	*M. latonigena*	BOLD:BPAL3476-16	**KY086170**	Russia	Altai	Pazhenkova and Lukhtanov 2016
*M. mauretanica*	*M. didyma*	NW107-5; BOLD:GBLN1855-09	**FJ462253**	Morocco		Leneveu et al. 2009
*M. mixta*	*M. chitralensis*	BOLD:MABUT253-11	**KC158427**	Pakistan	35.8333°N, 71.7667°E	Ashfaq et al. 2013
*M. mixta*	*M. chitralensis*	BOLD:MABUT254-11	**KC158426**	Pakistan	35.8333°N, 71.7667 °E	Ashfaq et al. 2013
*M. mixta*	*M. didyma*	BOLD:BPAL2509-14	**KT874722**	Tajikistan	Farob	Pazhenkova et al. 2015
*M. neera*	*M. neera*	BOLD:BPAL3482-16	**MW672084**	Kazakhstan	Zyryanovsk region, 49.62°N, 83.62°E	This study
*M. neera liliputana*	*M. didyma*	CCDB-17968 E10; BOLD:BPAL2718-14	**KT874744**	Israel	Hermon	Pazhenkova at al. 2015
*M. occidentalis*	*M. didyma*	RVcoll.08-L832	**GU676247**	Spain	Comunidad_de_Madrid	GenBank
*M. persea*	*M. persea*	BOLD:BPAL2349-14	**KY777522**	Iran	Tehran	Lukhtanov 2017
*M. persea paphlagonia*	*M. persea*	BOLD:BPAL2959-15	**KY777526**	Iran	Shahrud	Lukhtanov 2017
*M. saxatilis*	*M. saxatilis*	NW120-8; BOLD:GBLN1828-09	**FJ462281**	Iran	Tehran	Leneveu et al. 2009
*M. sutschana*	*M. sutschana*	BOLD:BPAL2543-14	**KT874696**	Russia	Chita	Pazhenkova et al. 2015
*M. telona*	*M. ornata telona*	BOLD:BPAL3126-15	**MW672062**	Israel		This study
*M. turkestanica*	*M. didyma*	BOLD:BPAL2770-15	**KY086115**	Kazakhstan	Saikan	Pazhenkova and Lukhtanov 2016

Sequences were aligned using the BioEdit software (Hall 1999) and edited manually. Phylogenetic hypotheses were inferred using Bayesian inference as described previously (Vershinina and Lukhtanov 2010; Przybyłowicz et al. 2014; Lukhtanov et al. 2016). Briefly, the Bayesian analysis was performed using the program MrBayes 3.2 (Ronquist et al. 2012) with default settings. Two runs of 10,000,000 generations with four chains (one cold and three heated) were performed. We checked runs for convergence and proper sampling of parameters [effective sample size (ESS) > 200] using the program Tracer v1.7.1 (Rambaut et al. 2018). The first 25% of each run was discarded as burn-in. The consensus of the obtained trees was visualized using FigTree 1.3.1 (http://tree.bio.ed.ac.uk/software/figtree/).

## Results

### Karyotype

**Figure 1.**
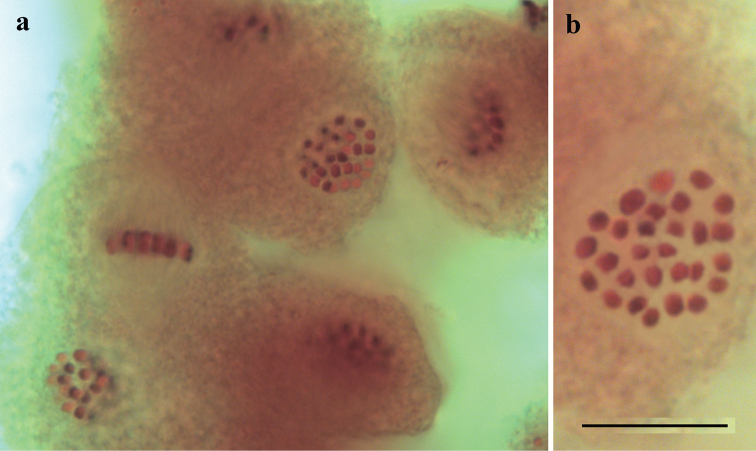
Karyotype of *M. kotshubeji***a** general view of six MI cells in a spermatocyst **b***M. kotshubeji*, AB080, MI, n=29. Scale bar: 10 μm.

The haploid chromosome number n=29 was found in prometaphase I, MI and MII cells of four studied individuals of *M. kotshubeji kotshubeji* (Table 2, Fig. 1). All chromosome elements formed a gradient size row. The karyotype contained no exceptionally large or small chromosomes.

**Table 2.** Chromosome number in studied samples of *Melitaea kotshubeji kotshubeji*.

Code number of the specimen	Chromosome number	Locality, date and collector	Number of cells checked
VLcoll.17-AB028	n=29	Tajikistan, Peter the Great Mts, Ganishou, 2200 m, 30.VI.2017, E. Pazhenkova leg.	5
VLcoll.17-AB080	n=29	Tajikistan, Peter the Great Mts, Muk, 2800 m, 25.VII.017, V. Lukhtanov leg.	7
VLcoll.17-AB086	n=29	Tajikistan, Peter the Great Mts, Muk, 2800 m, 26.VII.2017, V. Lukhtanov leg.	11
VLcoll.17-AB087	n=29	Tajikistan, Peter the Great Mts, Muk, 2800 m, 26.VII.2017, V. Lukhtanov leg.	14

### DNA barcode analysis

**Figure 2.**
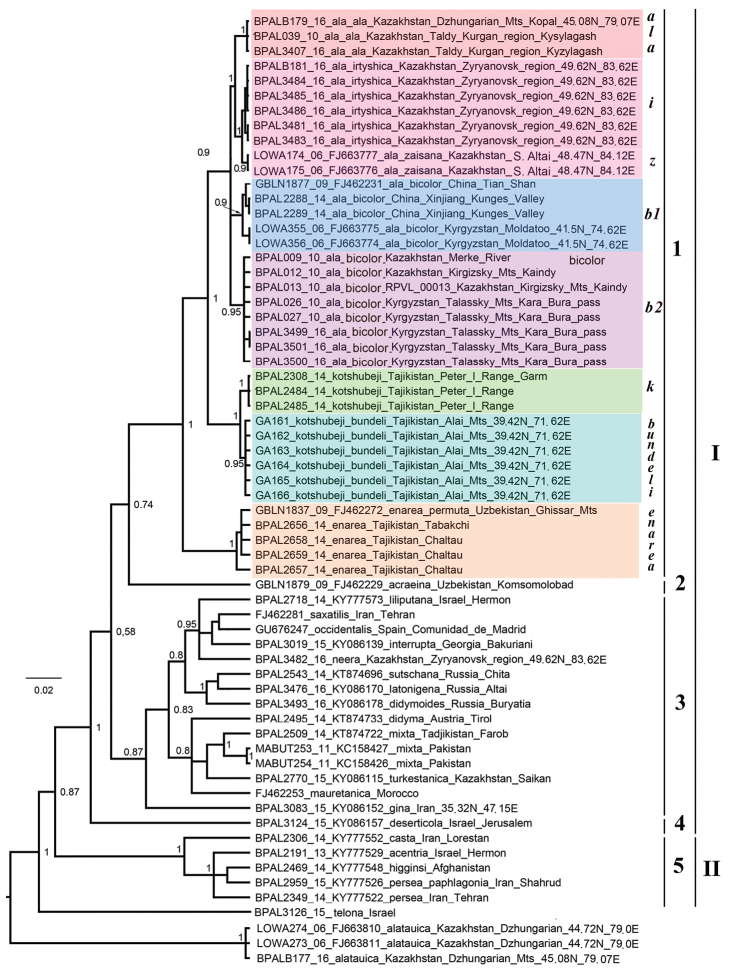
The Bayesian 50% majority rule consensus tree of the analyzed samples of *Melitaea* inferred from *COI* sequences. *Melitaea alatauica* and *M. telona* sequences are used to root the tree. Museum ID numbers, GenBank accession numbers, species and subspecies names, and localities are shown to the right of the branches. Bayesian posterior probabilities higher than 0.75 are shown next to the recovered branches. ***b1*** is *M. ala bicolor*, clade 1. ***b2*** is *M. ala bicolor*, clade 2. ***i****is M. ala irtyshica.****k*** is *M. kotshubeji kotshubeji*. ***z*** is *M. ala zaisana***1** is the *M. ala* species complex **2** is *M. acraeina***3** is the *M. didyma* species complex. **4** is *M. deserticola*. 5 is the *M. persea* species complex. I is *M. didyma* species group. II is *M. persea* species group.

DNA barcode analysis revealed *M. ala*, *M. kotshubeji* and *M. enarea* as highly supported monophyletic entities. Together, these three species formed a monophyletic lineage (the *M. ala* species complex) (1 in Fig. 2). In relation to the *M. ala* species complex, *M. acraeina* was found as a phylogenetically distant sister group (2 in Fig. 2). Taxa close to *M. didyma* (the *M. didyma* species complex) also formed a clade, but its support was relatively low (3 in Fig. 2). The species *M. deserticola* formed an independent lineage within the *M. didyma* species group (4 in Fig. 2). Together, these four lineages (*M. ala* complex + *M. acraeina* + *M. didyma* complex+ *M. deserticola*) formed the well-supported *M. didyma* species group (I in Fig. 2). The species of the *M. persea* group also formed a supported clade, sister to the *M. didyma* group (5 and II in Fig. 2).

Within the *M. ala* clade, five supported (Bayesian posterior probabilities ranged from 0.9 to 1.0), relatively weakly differentiated subclades were found. These are (1) *M. ala ala*, (2) *M. ala irtyshica*, (3) *M. ala zaisana*, (4) *M. ala bicolor* (clade *b1*) and (5) *M. ala bicolor* (clade *b2*). We also calculated the uncorrected *COI p*-distances within (Table 3) and between (Table 4) the revealed clades and groups.

**Table 3.** Intragroup uncorrected *COI p*-distances revealed within *M. ala*.

Group	Minimum p-distance	Maximum p-distance
irtyshica	0%	0.2%
zaisana	0%	0%
(irtyshica+zaisana)	0%	0.5%
ala	0%	0%
bicolor1	0%	0.6%
bicolor2	0%	0.2%
(bicolor1+bicolor2)	0%	0.8%

*Melitaea kotshubeji kotshubeji* and *M. kotshubeji bundeli* were found to differ by four fixed nucleotide substitutions in the *COI* barcode region.

**Table 4.** Uncorrected *COI p*-distances between the groups revealed within *M. ala*.

**Group 1**	**Group 2**	**Minimum p-distance**	**Maximum p-distance**
irtyshica	zaisana	0.3%	0.5%
(irtyshica+zaisana)	ala	0.9%	1.5%
(irtyshica+zaisana)	bicolor1	0.9%	1.5%
(irtyshica+zaisana)	bicolor2	0.9%	1.5%
ala	bicolor1	0.9%	1.3%
ala	bicolor2	0.9%	1.5%
bicolor1	bicolor2	0.3%	0.8%
(irtyshica+zaisana)	(bicolor1+bicolor2)	0.9%	1.5%
ala	(bicolor1+bicolor2)	0.9%	1.5%

## Discussion

### Chromosome number variation

The genus *Melitaea* (Fabricius, 1807) has relatively low interspecific chromosome number variation. The representatives of basal clades (see phylogeny in Leneveu et al. 2009), the taxa of *M. cinxia* (Linnaeus, 1758), *M. diamina* (Lang, 1789), *M. athalia* (Rottemburg, 1775), *M. trivia* ([Denis et Schiffermüller], 1775) and *M. phoebe* ([Denis et Schiffermüller], 1775) species groups demonstrate n=30–31 (Federley 1938; de Lesse 1960; Robinson 1971; Larsen 1975; Hesselbarth et al. 1995). These haploid numbers are modal ones not only for *Melitaea*, but also for the family Nymphalidae and for the order Lepidoptera in whole (Robinson 1971; Lukhtanov 2000, 2014). Most likely, one of them (probably, n=31, see Lukhtanov 2014) represents an ancestral lepidopteran state preserved in the basal lineages of *Melitaea*.

The *Melitaea didyma* species group is one of the younger lineages of *Melitaea* (Leneveu et al. 2009). This group is found to have lower chromosome numbers varying from n=27 to n=29–30 (Table 5). *Melitaea didyma* species complex is characterized by chromosome numbers from n n=27 to n=30, with n=28 and n=29 as modal numbers. In the *Melitaea deserticola* species complex, only one species (*M. deserticola*) is karyotyped (n=29). In the *Melitaea persea* species complex, n=27 is found in two species. In the *Melitaea ala* species complex, n=29 is found in two species studied.

**Table 5.** Chromosome nmbers of taxa close to *M. didyma*.

Species complex	Taxon	Chromosome number	Country	Locality	Reference
*Melitaea didyma* species complex	*M. didyma*	n=28	Italy	Abruzzi	de Lesse 1960
	*M. didyma neera*	n=28	Kazakhstan	Altai	Lukhtanov and Kuznetsova 1989
	*M. didyma neera*	n=27	Russia	N Caucasus, Pyatigorsk	Lukhtanov and Kuznetsova 1989
	*M. interrupta*	n=29	Turkey		de Lesse 1960
	*M. interrupta*	n=29	Azerbaijan, Nakhichevan	Zangezur Mts	Lukhtanov and Kuznetsova 1989
	*M. latonigena*	n=29–30	Kazakhstan	Altai	Lukhtanov and Kuznetsova 1989
	*M. gina*	n=28	Iran	W Azerbaijan	Pazhenkova and Lukhtanov 2016
*Melitaea deserticola* species complex	*M. deserticola*	n=29	Lebanon		Larsen 1975
*Melitaea ala* species complex	*M. ala*	n=29	Kazakhstan		Lukhtanov and Kuznetsova 1989
	*M. kotshubeji*	n=29	Tajikistan		This study
*Melitaea persea* species complex	*M. persea*	n=27	Iran		de Lesse 1960
	*M. acentria*	n=27	Israel		Lukhtanov 2017

Based on the distribution of the known chromosome numbers (Table 3) relative to the phylogeny (Fig. 2) and on the frequency of their occurrence, we can assume that n=29 is an ancestral state for the species of the *M. didyma* group. Thus, for the species of the *M. ala* complex n=29 is a symplesiomorphy.

### Intraspecific taxonomy of the *M. ala* species group

The five identified clades within the species *M. ala* have relatively high support (Fig. 2) and can be considered as taxa, at least from the standpoint of the phylogenetic species concept (Cracraft 1989; Coyne and Orr 2004), in which diagnosable entities can be classified as species regardless of whether there is reproductive isolation between them or not. To assess the possibility of interpreting these clades as species or subspecies, we compared the level of *COI* divergence between the clades with the level of variability within the clades (Tables 3, 4). We found that in all cases, the distances between these clades were lower than ‘standard’ DNA-barcode species threshold (3%) (Hebert et al. 2003).

An especially low level of differentiation (0.3–0.5%) was found between the clades *M. ala zaisanica* and *M. ala irtyshica*. Therefore, we are inclined, especially taking into account the geographical proximity of these lineages, to consider them as a single taxonomic unit, *M. ala zaisanica* (= *M. ala irtyshica*).

A slightly higher average level of differentiation (0.3–0.8%) was found between the *b1* and *b2* clades (Fig. 2, Table 4). However, in this case, a rather high level of intragroup variability was observed (Table 3), and the maximum values of intragroup variability exceeded the minimum intergroup differences. Therefore, taking into account the geographical proximity of these lineages, we decided to consider them as a single taxonomic unit, *M. ala bicolor*.

Thus, within the studied populations, three subspecies can be distinguished. These are *M. ala ala*, *M. ala bicolor* and *M. ala zaisana*.

**Figure 3.**
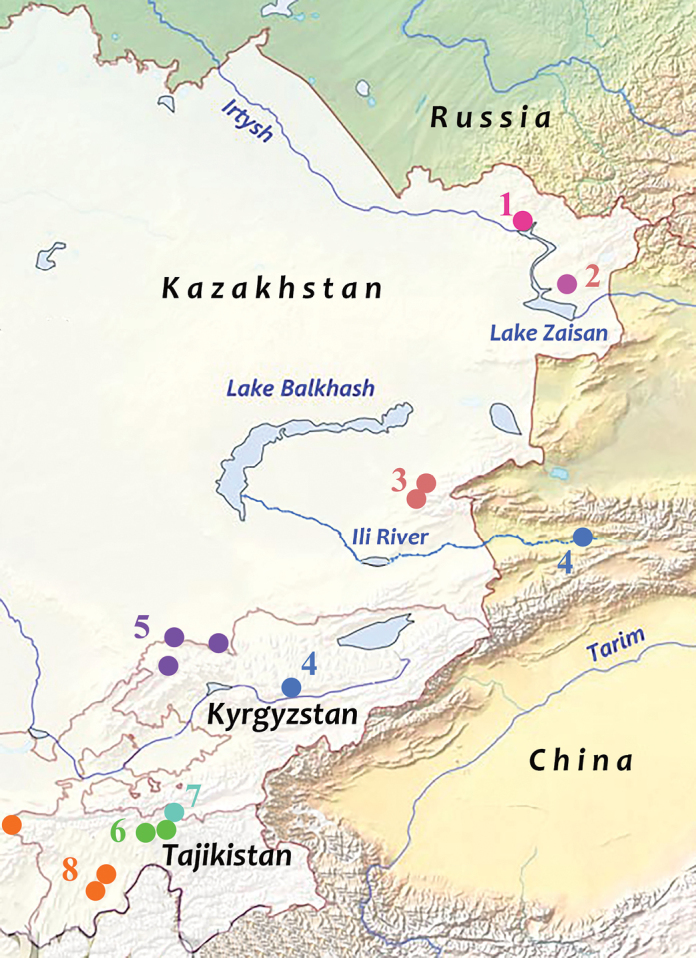
Locations of the analyzed samples of *M. ala*, *M. kotshubeji* and *M. enarea***1** type-locality of *M. ala irtyshica* (Kazakhstan, Zyryanovsk district, Oktyabrsk, 49.62°N, 83.62°E) **2** type-locality of *M. ala zaisanica* (Kazakhstan, Kurtchumski Mts, 48.47°N, 84.12°E) **3***M. ala ala* (Kazakhstan, Dzhungarian Alatau, Kyzylagash and Kopal) **4***M. ala bicolor* (clade *b1*) (China, Kyrgyzstan) **5***M. ala bicolor* (clade *b2*) (Kyrgyzstan, Kara-Bura Pass; Kazakhstan, Kirgizski Mts) **6***M. kotshubeji kotshubeji* (Tajikistan, Peter the Great Mts) **7***M. kotshubeji bundeli* (Tajikistan, border with Kyrgyzstan, Alai Mts, 39.42°N, 71.62°E) **8***M. enarea* (Tajikistan).

**Figure 4.**
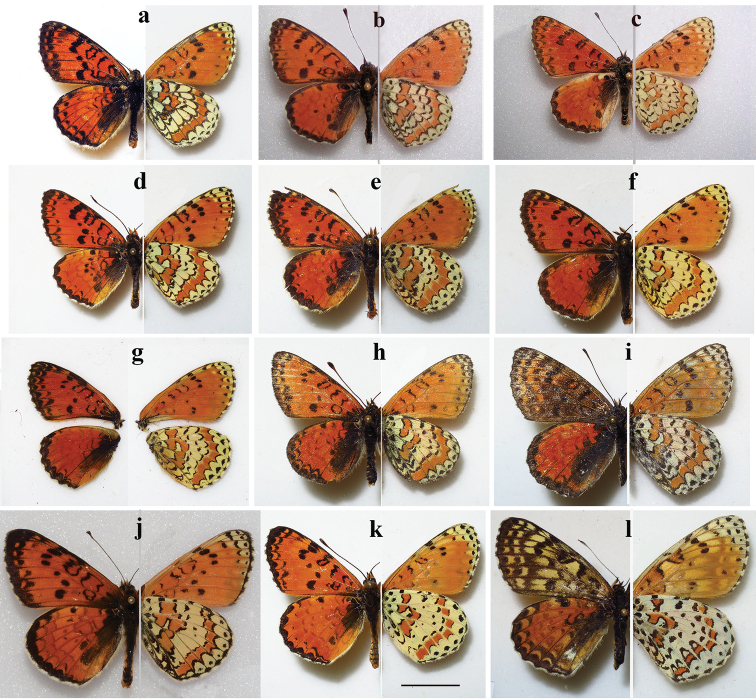
Butterflies of the *Melitaea ala* species complex **a***M. ala ala*, male, BPALB179-16 (CCDB-25458_G12), Kazakhstan, Dzhungarian Alatau, Kopal, 45.04°N, 79.06°E, 1800–1900 m, 13.VI.2016, V. Lukhtanov leg. **b***M. ala bicolor*, clade *b1*, male, Kyrgyzstan, Moldatoo Mts, 41.5°N, 74.62°E, 2100 m, 29.VI.1996, V. Lukhtanov leg. **c***M. ala zaisana*, male, LOWA174-06, Kazakhstan, Kurchumski Khrebet, Kalgutinski Pass, 600 m, 48.47°N, 84.12°E, 9.VI.1998, V. Lukhtanov leg. **d***M. ala irtyshica*, male, BPAL3484-16 (CCDB-25456_F04), Kazakhstan, Zyryanovsk distr., Oktyabrsk, 49.6178°N, 83.6219°E, 420 m, 08.VI.1999, V. Lukhtanov leg. **e***M. ala bicolor*, clade *b2*, male, CCDB-03024-RPVL-00009, Kazakhstan, Kirgizski Mts, Merke, 42.69°N, 73.25°E, 1500m, 13.VI.2000, V. Lukhtanov leg. **f***M. ala bicolor*, clade *b2*, male, BPAL027-10 (RPVL-00027), Kyrgyzstan, Talassky Mts, Kara-Bura pass, 42.27°N, 71.57°E, 2000m, 30.VI.2000, V. Lukhtanov leg. **g***M. ala bicolor*, clade *b2*, male, BPAL026-10 (RPVL-00026), Kyrgyzstan, Talassky Mts, Kara-Bura pass, 42.27°N, 71.57°E, 2000m, 30.VI.2000, V. Lukhtanov leg. **h***M. kotshubeji bundeli*, male, GA161, Tajikistan, Alai Mts, Kichi-Karamuk, 39.4258°N, 71.6125°E; 3150 m, 03.VIII.2019, V. Lukhtanov leg. **i***M. kotshubeji bundeli*, female, GA166, Tajikistan, Alai Mts, Kichi-Karamuk, 39.4258°N, 71.6125°E; 3150 m, 03.VIII.2019, V. Lukhtanov leg. **j***M. kotshubeji kotshubeji*, male, BPAL2484-14 (CCDB-17966 B02), Tajikistan, Peter I Range, 7 km S Tajikobad, 14.VIII.2003 **k***M. enarea*, male, BPAL2656-14 (CCDB-17967_H07), Tajikistan, Tabakchi Mts, 37.85°N, 68.98°E, 1150 m, 01.V.2014, V. Lukhtanov leg. **l***M. enarea*, female, BPAL2659-14 (CCDB-17967_H10), Tajikistan, Chaltau Mts, 37.9550°N, 69.1403°E, 1041m, 02.V.2014, V. Lukhtanov leg. Scale bar: 10 mm

*Melitaea ala ala* is distributed in the Dzhungarian Alatau in East Kazakhstan (Fig. 3). This subspecies is characterized by darkening of the veins on the underside of the hind wing. These darkened veins form clear cells in the region of the median band (Fig. 4a).

*Melitaea ala bicolor* Seitz, 1908 is distributed in the North, East, Central and West Tian-Shan in SE Kazakhstan, NW China and Kyrgyzstan (Fig. 3). In this subspecies the veins on the underside of the hind wing are not strongly darkened. The cells of the median band are not highlighted. They are only marked with dark brackets on the outside of the median band (Fig. 4b). The specimens from the Tyshkantau Mts (SE part of the Dzhungarian Alatau in Kazakhstan) (Tuzov and Churkin 2000) and the eastern most part of the Tian-Shan (Kolesnichenko 1999) are intermediate between *M. ala ala* and *M. ala bicolor*.

With regards to DNA barcodes, *M. ala zaisana* Lukhtanov, 1999 (Fig. 4c) is distinct from the geographically closest *M. ala ala*. With regards to the wing pattern, *M. ala zaisana* is more similar to *M. ala bicolor* than to *M. ala ala*. Interestingly, the northernmost population of *M. ala* from Oktyabrsk (Kazakhstan) (Fig. 3d) is intermediate in its appearance between *M. ala ala* and *M. ala zaisana*. This population was described as *M. ala irtyshica* Lukhtanov, 1999 (Lukhtanov 1999) and was later erroneously synonymized with *M. latonigena* Eversmann, 1847 (Lukhtanov et al. 2007). DNA barcode analysis demonstrates that this population is similar to *M. ala zaisana*.

**Figure 5.**
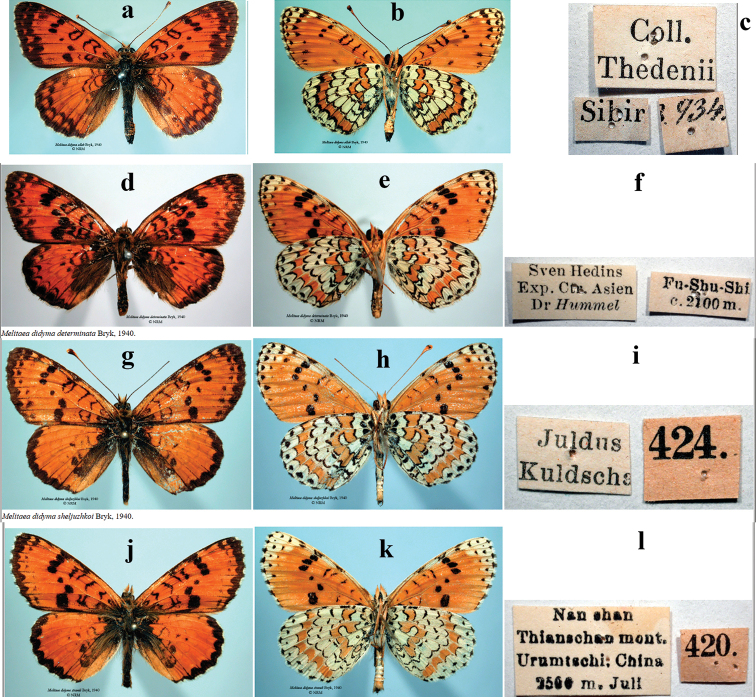
Syntypes of the taxa of the *Melitaea ala* species complex, originally described by Felix Bryk (1940) as subspecies of *M. didyma*. All specimens are deposited in Swedish Museum of Natural History (Naturhistoriska riksmuseet, NRM) **a***M. didyma allah*, upperside **b***M. didyma allah*, underside **c***M. didyma allah*, labels **d***M. didyma determinata*, upperside **e***M. didyma determinata*, underside **f***M. didyma determinata*, labels **g***M. didyma sheljuzhkoi*, upperside **h***M. didyma sheljuzhkoi*, underside **i***M. didyma sheljuzhkoi*, labels **j***M. didyma strandi*, upperside **k***M. didyma strandi*, underside **l***M. didyma strandi*, labels.

Currently, there is a tendency to consider as a species any group of populations with a minimum set of fixed differences. We are almost certain that, given this trend, the subspecies discussed above will be interpreted by some authors as species in the future. Nevertheless, in our opinion, in accordance with the subspecies criteria (Lukhtanov et al. 2016; De Queiroz, 2020), they should be treated as subspecies of the same species.

*Melitaea kotshubeji bundeli* (Fig. 4h, i) was described as subspecies of *Melitaea kotshubeji* (Fig. 4j) (Kolesnichenko 1999), but later was treated as a distinct species (van Oorschot and Coutsis 2014) or alternatively as a synonym (Tshikolovets 2003, 2005). Our study demonstrates that these two taxa are not only distinct in the wing pattern, but also differ by four fixed nucleotide substitutions in the DNA barcode region, indicating the relative long independent evolution of these two sublineages. Interestingly, the distribution areas of these two allopatric taxa are in close proximity to each other and are separated by a narrow valley of the Surkhob River (in Kyrgyzstan, this river is called the Kyzylsu).

In our work we do not consider the intraspecific structure of *M. enarea* (Fig. 4k, l) due to the lack of molecular data for the northern populations of this species.

### The taxa described by Bryk (1940)

Bryk (1940) described four taxa (all as subspecies of *M. didyma*) that should be assigned to *M. ala*. The types of these taxa were studied by the first author of this article in 2007 during a visit to Swedish Museum of Natural History.

The taxon described by Bryk (1940) as *M. didyma allah* Bryk, 1940 has the wing pattern with clear characters of *M. ala ala* (Fig. 5a, b), but not of the subspecies *M. ala zaisana* (Fig. 4c) as supposed by Tuzov and Churkin (2000). Thus, *M. didyma allah* should be synonymized with *M. ala ala* as suggested by Kolesnichenko (1999). We agree with Kolesnichenko (1999) that the label data of the syntype of *M. didyma allah* (Fig. 5c) are probably wrong.

The taxa described by Bryk (1940) as *M. didyma sheljuzhkoi* Bryk, 1940 (Fig. 5g–i) and *M. didyma strandi* (Fig. 5j–l) have the wing pattern with characters of *M. ala bicolor*. Most likely, they represent synonyms of *M. ala bicolor*.

The taxon from “Fu-Shu-Shi” (China) described by Bryk (1940) as *M. didyma determinata* Bryk, 1940 is characterized by the well-developed black wing pattern on both wing upper- and underside (Fig. 5d–f). Most likely, it represents a distinct subspecies. Unfortunately, we do not have material for molecular study to test this hypothesis.

### Probably erroneous species identifications in the *M. ala* complex

The specimens identified as *Melitaea ninae* (sample NW113-10, FJ462269, Kyrgyzstan), *M. enarea* (sample NW113-15, FJ462256, Tajikistan) (Leneveu et al. 2009; Long et al. 2014) and *M. chitralensis* (samples KC158426 and KC158427) (Ashfaq et al. 2013) were reported in the cited molecular phylogenetic analyses of the genus *Melitaea*. According to the DNA barcodes of these samples, they most likely belong to *M. turkestanica* Sheljuzhko, 1929 (NW113-10) and *M. mixta* Evans, 1912 (NW113-15, KC158426 and KC158427).
